# Am I Happier Without You? Social Media Detox and Well-Being: A Meta-Analysis of Randomized Controlled Trials

**DOI:** 10.3390/bs15030290

**Published:** 2025-03-01

**Authors:** Yuyang Liu, Emma Mirza Wati Mohamad, Arina Anis Azlan, Yunpeng Tan

**Affiliations:** 1 Centre for Research in Media and Communication, Faculty of Social Sciences and Humanities, Universiti Kebangsaan Malaysia, Bangi 43600, Malaysia; p129558@siswa.ukm.edu.my (Y.L.); arina@ukm.edu.my (A.A.A.); 2 School of Fashion Media, Jiangxi Institute of Fashion Technology, Nanchang 330201, China; 3 Komunikasi Kesihatan (Healthcomm)-UKM Research Group, Universiti Kebangsaan Malaysia, Bangi 43600, Malaysia; 4 School of Journalism and Communication, Xiamen University, Xiamen 361005, China; mixtyp@163.com

**Keywords:** social media detox, well-being, randomized controlled trials, meta-analysis

## Abstract

With the penetration of social media into all aspects of people’s lives, there is a growing trend of social media detox (taking a short break from social media). Although social media detox (SMD) has been theorized to influence well-being, vibrant research on this issue presents mixed results. This raises the question of whether SMD has positive or negative effects on well-being, calling for a synthesis of empirical evidence to determine if engaging in SMD can improve well-being. Systematic review and meta-analysis were conducted to synthesize evidence on the relationship between SMD and well-being from randomized controlled trials (RCTs). A total of 20 RCTs and 56 effect sizes (positive indicators of well-being k = 33; negative indicators of well-being k = 23) were analyzed. The meta-analysis findings using random effects showed that there was a positive and small effect of SMD on well-being, including positive indicators and negative indicators. The results of the moderating analysis showed that there was heterogeneity in the relationship between SMD and well-being caused by cultural background. Moreover, intervention duration moderated the relationship between SMD and negative indicators of well-being. This meta-analysis allows for an integration of conclusions from past studies and establishes a clearer understanding of the relationship between SMD and well-being.

## 1. Introduction

The rapid advancement of the Internet, coupled with the ongoing evolution of smart and mobile devices, has rendered social media an essential tool for communication and entertainment in contemporary society. However, as social media becomes increasingly woven into daily life, users are confronted with the challenges of message overload, excessive connectivity, privacy concerns, and social media fatigue, which can lead to negative usage behaviors (Dhir et al., 2019; Feng et al., 2024; Maier et al., 2015; Zhang et al., 2016). Consequently, users are gradually transitioning from social media addiction to a practice known as social media detox (SMD). Scholarly attention has also shifted from social media use, social media consumption, and social media addiction to social media detox (Efimova & Semenov, 2020; Purohit et al., 2020; Scheppe & Seiffen, 2022). However, the extent to which SMD can positively influence individuals’ overall well-being remains unclear. While much research has explored the effects of SMD on well-being, the results are inconsistent and lack a definitive conclusion. While some studies report positive correlations between SMD and the positive indicators of well-being, such as life satisfaction, self-esteem, and perceived social support (Chen et al., 2022; Hanley et al., 2019; Reinecke & Oliver, 2016; Schwarz et al., 2022), other research has linked SMD to adverse outcomes such as loneliness and stress (Stieger & Lewetz, 2018; Vally & D’Souza, 2019). Although a systematic literature review conducted by Sharma et al. (2020) summarized the mental health impact of social media, their analysis was limited to the positive aspects of social media use. Further analysis of negative use (e.g., social media detox) is needed. Additionally, Radtke et al. (2022) provided a qualitative literature review of digital detox aimed at enhancing health and well-being, yet their focus was primarily on smartphone detox rather than SMD. Therefore, it is essential to employ more robust methodologies to quantify the strength of the relationship between SMD and well-being.

Given these considerations, a meta-analysis is essential to investigate the relationship between SMD and well-being while also exploring potential moderating factors. This study aims to (1) assess the associations between various forms of SMD and well-being by synthesizing the findings from previous randomized controlled trials; (2) analyze both the positive and negative indicators of well-being independently, thereby providing a more precise evaluation of their relationship with SMD; (3) explore the influence of several moderators on the connection between SMD and well-being; and (4) identify critical areas that need further research.

### 1.1. Social Media Detox and Well-Being

Social media detox (SMD) extends from the concept of digital detox. Officially recognized in the Oxford Dictionary since 2013, “digital detox” is defined as “a period of time when a person refrains from using electronic devices such as smartphones or computers, regarded as an opportunity to reduce stress or focus on social interactions in the real world”. SMD entails users consciously refraining from using and consuming social media for a predetermined period (Turel, 2015; Turel & Vaghefi, 2019). Additionally, various other terms are utilized to describe this practice, including abstinence, time out, taking a break, discontinuance, and unplugged (Fioravanti et al., 2020). For this study, all forms of discontinuous social media usage behavior are categorized as SMD.

Well-being is considered to be the ultimate driving force of human activity, and scholarly interest in well-being has persisted over time. Well-being is defined as the integration of feeling good and performing well (Huppert, 2009). Well-being serves not only as a reflection of the social status emphasized by many countries but also as a crucial indicator of mental health (Sacks et al., 2010). According to Houben et al. (2015), the construct of well-being can be approached from various perspectives, including positive indicators (e.g., hedonic, eudaimonic, and social well-being) and negative indicators (e.g., anxiety, loneliness, and depression). It is noteworthy that positive and negative indicators should not be viewed as strictly oppositional, as they may involve distinct components (Yin et al., 2019). This study undertook a thorough evaluation of various well-being indicators, examining positive and negative aspects separately, to elucidate the relationship between SMD and outcome variables with greater clarity and precision.

### 1.2. Previous Outcomes of Studies Related to Social Media Detox and Well-Being

Research has increasingly focused on the intricate relationship between SMD and overall well-being. Various scholars have highlighted the advantages of SMD in their investigations. For instance, Shakya and Christakis (2017) revealed that regulating Facebook usage can enhance positive emotions. Similarly, Stieger and Lewetz (2018) conducted an experiment demonstrating that restricting Facebook, Instagram, and Snapchat usage to 10 min for four weeks resulted in decreased levels of depression and loneliness. A short break from Facebook lasting between 2 and 7 days can increase users’ life satisfaction (Hall et al., 2019), while capping social media engagement at about 30 min a day decreases loneliness and depressive symptoms (Hunt et al., 2018). Conversely, some studies have indicated that SMD may negatively influence users’ well-being. Vally and D’Souza (2019) observed that abstaining from social media can result in lower levels of life satisfaction, as well as heightened negative emotions and feelings of loneliness when compared to a control group. Many users, particularly college students, passively engage with social media due to fear of missing out (Alt, 2015). However, SMD may intensify this fear, leading to negative affects, such as anxiety (O’Connell, 2020). Furthermore, several studies have shown that SMD does not change well-being (Agadullina et al., 2021; Hall et al., 2021; Przybylski et al., 2021).

Previous research has employed various theoretical frameworks and mechanisms to elucidate the relationship between SMD and well-being. According to a presumption of balance, Syvertsen and Enli (2020) suggested that SMD promotes a balance between users’ offline and online lives as a response to improved health and mindful presence while experiencing temporary overload and intrusion. Additionally, based on the underlying cognitive and behavioral mechanisms, Zhou et al. (2021) pointed out that SMD protects the user from distractions and enables them to concentrate on other significant activities, so the user has a positive feeling and a perceived sense of accomplishment. Further, by combining the Communicate Bond Belong theory and related conceptualizations of digital solitude, Nguyen and Hargittai (2024) showed that people invest their social energy in beneficial social interactions, and SMD is prioritized for face-to-face social interaction, which may promote connection and well-being overall.

This study is grounded in the Uses and Gratifications Theory (UGT), originally proposed by Elihu Katz in 1959. UGT has proven to be a valuable framework for examining the interplay between social media and well-being. For instance, researchers have investigated the motivations behind social media use among housewives, identifying five primary drivers: escapism, information seeking, friendship maintenance, relationship building, and nostalgia (Hamid et al., 2022). In addition, Jo and Jang (2023) analyzed how adolescents utilize social media to forge connections and enhance their well-being, offering clear recommendations for responsible social media engagement. In contrast to prior research, this meta-analysis focused on the phenomenon of SMD, a form of “no-use” behavior, seeking to analyses whether and what kind of gratifications are associated with this “no-use” behavior (SMD). And, many studies that focused on SMD used well-being as an important outcome of gratifications (Hughes & Burke, 2018; Tromholt, 2016; Vanman et al., 2018). Therefore, we developed the following research hypotheses to further investigate the nuanced relationship between SMD and well-being:

**H1.**  *Overall, social media detox affects well-being.*

**H1a.**  *Social media detox affects the positive indicators of well-being. (In other words, social media detox increases the positive feelings to improve well-being.)*

**H1b.**  *Social media detox affects the negative indicators of well-being. (In other words, social media detox reduces the negative feelings to improve well-being.)*

### 1.3. Potential Moderators

Previous empirical research examining the intricate relationship between SMD and users’ well-being has produced inconsistent findings, potentially due to the moderating effects of various participant and situational factors. Based on a comprehensive review of the existing literature, five variables such as age, gender, cultural background, detox types, and duration are selected for potential moderating effect testing.

Previous research has shown that well-being levels vary across age groups (Blanchflower & Oswald, 2008; Dardha & Rogge, 2020). Steptoe et al. (2015) pointed out that well-being is related to many factors such as social and family relations, social roles, and activities, which change with age. And users of different ages have different perceptions and motivations for using social media. Research has shown that teenagers prefer to make new friends and post more frequently and rapidly, while older people prefer to write longer posts and discuss family matters more (Chang et al., 2015; Pfeil et al., 2009). Research has also suggested that the adverse effects of social media overload become more pronounced as users age (Zhang et al., 2016). Consequently, SMD might exert a greater effect on older users. Age should, therefore, be investigated as a potential moderator, and we proposed the following research hypothesis:

**H2.**  *A stronger effect of social media detox on well-being would be observed among older samples.*

Besides age, gender should be considered when analyzing the relationship between SMD and well-being. Gender groups differ in their use and reliance on social media. Research indicates that female users generally allocate more time to social media than male users and often have larger online networks of friends on these platforms (Theophilou et al., 2024; Vošner et al., 2016). Compared to men, women are more likely to exhibit social media addiction (Kong et al., 2023; Su et al., 2020). Research by Zhang et al. (2016) that explored social media discontinuous use behavior pointed out that males are more prone to feelings of fatigue and tiredness associated with social media use than females. Further, males and females have different attitudes and outcomes regarding SMD: females have more positive attitudes and better outcomes than males (Fioravanti et al., 2020). Considering these gender differences, it is essential to investigate gender as a potential moderator. Accordingly, we proposed the following research hypothesis:

**H3.**  *A stronger effect of social media detox on well-being would be observed in samples with a greater proportion of female participants.*

Many studies have found that individualism–collectivism orientations are related to people’s well-being, but the relationship between them is still controversial. Some scholars hold the opinion that both of these orientations are correlated with personal well-being (Novin et al., 2014; Suh et al., 2008), while others argue that people with a more individualistic orientation report greater personal well-being (Park et al., 2017; Yamaguchi & Kim, 2015). Conversely, other researchers have established a positive correlation between collectivism and well-being (Germani et al., 2021; Nezlek & Humphrey, 2023; Zalewska & Zawadzka, 2016).

Furthermore, individualists are influenced by their feelings and cognition, which are usually not affected by others, and those with individualistic orientations actively strive for their status, hoping to realize their self-value through self-reliance. On the contrary, those with collectivist tendencies prioritize the needs, desires, and achievements of their group or organization over their own, often aligning their personal goals with the objectives of the collective (Triandis, 2001). Thus, people living in a collectivist social background tend to prioritize their connection with the collective, which can lead to social overload that causes anxiety and depression. SMD might, therefore, alleviate these negative emotions. We hypothesize that the relationship between SMD and well-being will be more pronounced in collectivist cultures compared to individualistic ones. As mixed culture involves the characteristics of the two cultures, the effect of a mixed culture background would be between the two. According to Hofstede’s cultural dimension scores, we divided participants’ cultural backgrounds by their ethnicities into individualism, collectivism, and mixed culture. And, we proposed the following research hypothesis:

**H4.**  *The effect of social media detox on well-being would likely be stronger in collectivism countries compared to individualism countries and mixed culture countries.*

In intervention experiments exploring the relationship between SMD and well-being, a variety of intervention types are employed. Full abstinence is the most common type, which asks participants not to use social media at all for a specified duration (Przybylski et al., 2021; Vally & D’Souza, 2019). There is another type of intervention that is not full abstinence but involves placing restrictions and limitations on social media use, such as time limitations (Brailovskaia et al., 2020; Hunt et al., 2021). Some studies use therapy-based interventions for SMD (Chen et al., 2022; Nguyen & Hargittai, 2024). Therapy-based intervention involves using therapeutic techniques, including group psychological counseling or cognitive behavioral therapy (CBT), to facilitate reflection on social media-related habits, thoughts, and emotions while also promoting effective time management strategies (Plackett et al., 2023). A study compared social media reduction with social media abstinence and found that reduction had a better effect on well-being and a healthy lifestyle than abstinence (Brailovskaia et al., 2023). Further, some studies argued that interventions focused simply on restricting social media use or enforcing full abstinence may provide less benefit for mental well-being compared with therapy-based methods (Plackett et al., 2023). Thus, this study classified the types of SMD interventions into full abstinence, limited use, and therapy-based interventions, and we proposed the following research hypothesis:

**H5.**  *Among the three types of social media detox (full abstinence, limited use, and therapy-based interventions), the effect size of therapy-based interventions on well-being was the largest.*

Besides the types of detox, the duration of these interventions may serve as a significant moderator in the correlation between SMD and well-being. Most studies focused on short-term interventions, such as one or two weeks (Schwarz et al., 2022; Vally & D’Souza, 2019), but there were also studies that lasted two months (Collis & Eggers, 2022). As abstinence continued, the negative effects decreased as participants slowly adjusted to abstinence (Zhou et al., 2021). Thus, overall well-being may grow with the duration of the SMD. Based on this reasoning, the following research hypothesis was proposed:

**H6.**  *A stronger positive effect of social media detox on well-being would be observed among samples with a longer detox duration.*

## 2. Materials and Methods

### 2.1. Literature Search

A systematic literature search was conducted on 30 June 2024 across multiple databases, including Web of Science/SSCI, Social Science Database, Springer, PsycINFO, Elsevier SDOL, and Google Scholar. The search utilized comprehensive combinations of keywords related to social media (e.g., social network site, social networking site SNS, online, Facebook, Instagram, Twitter, Myspace, WeChat, TikTok, DouYin, Qzone, and Weibo) detox (e.g., detoxication, negative use, discontinuance, discontinuous use, no-use, limited use, intervention, take a break, abstinence, time out, quitting, unplugged, reduction, and restriction), and well-being (e.g., mental health, life satisfaction, positive and negative affects, emotion, feeling, social support, self-esteem, depression, loneliness, anxiety, envy, and stress). Additionally, in order to avoid missing unpublished gray literature, we consulted the dissertations libraries of universities and academic institutions, such as ProQuest Dissertations and Theses, and gray literature databases, such as OpenGrey and Grey Literature Report. We also reviewed the abstracts of academic conferences and tried to contact relevant authors for unpublished data.

**Figure 1:**
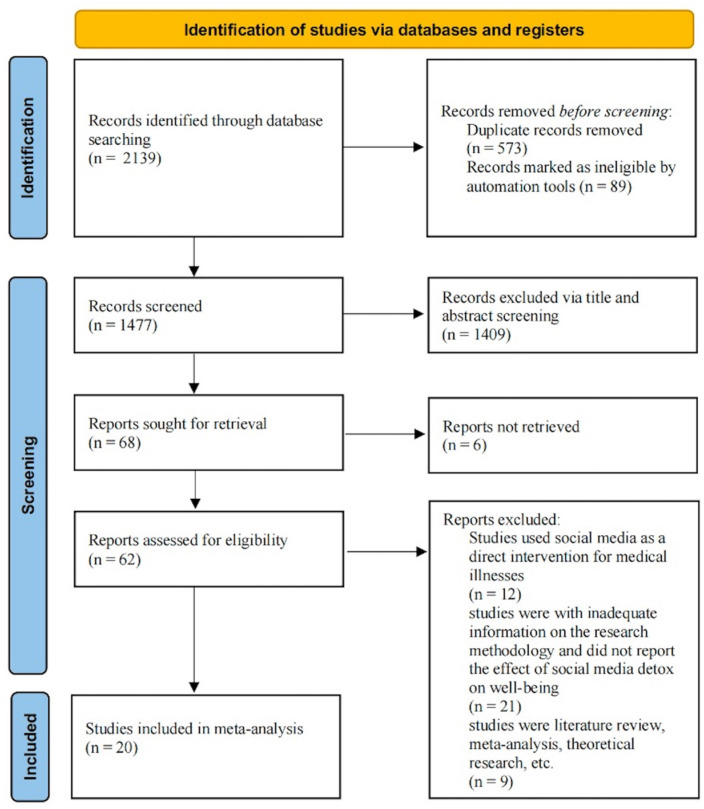
PRISMA flow diagram of study selection process.

This initial search yielded a total of 2319 results. After removing duplicates, the titles and abstracts of the remaining 1477 articles were screened to identify randomized controlled trials that examine the relationship between SMD and well-being. Ultimately, 68 articles were selected for inclusion based on this screening process, and we downloaded the full texts of these literature for further examination. Details of this process follow the PRISMA protocol as outlined in Figure 1.

### 2.2. Inclusion and Exclusion Criteria

Only studies that met the following criteria were selected for inclusion:

Type of studies: (a) English was the publication language; (b) the publication was a study rather than a news report; (c) the study conducted a randomized controlled trial (RCT); (d) the study investigated SMD intervention characterized by voluntary limited absence from use; and (e) inferential statistics were employed to assess the intervention’s effects in comparison to a control group.

Type of sample: (a) samples were drawn from general populations, excluding those focused on specific groups (such as intellectually disabled, physically disabled, intellectually gifted, etc.); (b) studies featuring two or more independent samples were analyzed separately for this meta-analysis.

Type of outcome: (a) dependent variables included measures of well-being, such as life satisfaction, self-esteem, social support, depression, loneliness, anxiety, stress, positive affect, or negative affect; (b) effect sizes were reported, or enough data were available to calculate them.

Exclusion criteria: (a) studies that utilized social media as an intervention for medical conditions; (b) studies with insufficient methodological details or those that did not report the effects of SMD on well-being; (c) studies that were literature reviews, meta-analyses, theoretical research, etc.; and (d) research data that were duplicated.

Following a thorough review of all the full texts, there were a total of 42 articles excluded, resulting in a preliminary dataset comprising 20 articles (as shown in the Supplementary Materials Table S1). A summary of the selection process, consistent with the PRISMA protocols (Moher et al., 2015), is presented in Figure 1.

### 2.3. Data Extraction

Data were extracted using a structured table in Excel (Microsoft Corp, Redmond, Washington, USA) to capture the following information: (a) authors, (b) publication years, (c) country, (d) number of participants, (e) age, (f) proportion of female participants, (g) detox types, (h) detox duration, (i) platforms of interventions, (j) indicators of well-being, and (k) effect size. To ensure accuracy and consistency, a second reviewer verified the extraction of data from the full-text articles. The data were shown in the Supplementary Materials Table S2.

### 2.4. Coding

Potential moderator variables were systematically coded, including age, gender, culture, detox types, and detox duration. Age was coded according to the mean age of the participants. Gender was coded according to the proportion of female participants. The cultural background was coded as (1) individualism (including the United States, the United Kingdom, Germany, Denmark, Australia, and Norway), (2) collectivism (including China and the United Arab Emirates), and (3) mixed culture (including Italy). One article did not explicitly state the ethnicity of the participants, so it was excluded for this type of coding. SMD intervention types were coded as (1) full abstinence, (2) limited use, and (3) therapy-based intervention. The intervention duration was coded based on the length of the intervention. Throughout the data coding process, independent studies were used as the coding unit. If there were multiple effect values from different samples in one study, multiple coding was carried out to obtain multiple effect values. Three researchers initially coded the included literature according to the coding steps outlined by Lipsey and Wilson (2001), with each researcher working independently. Next, the coding results were then cross-checked among the three researchers, resulting in a consistency ratio of 85.9%. This ratio indicated a high level of agreement but highlighted the existence of some discrepancies that required further discussion and resolution. Multiple meetings were held for discussion, where the researchers reviewed each code that exhibited discrepancies. For specific cases that were contentious, researchers engaged in deep discussions about the coding rationale. For instance, in coding for cultural background, differing views could arise regarding the classification of certain countries. Researchers referred to the relevant literature and theories (Hofstede & Bond, 1984) to determine whether specific countries aligned more with individualism, collectivism, or mixed culture. After further discussion and review, a consensus was reached by revising the parts where discrepancies were identified.

### 2.5. Publication Bias

It is necessary to assess for any indication of potential publication bias, as such bias can result in an inflated estimation of effect sizes. This occurs because empirical studies, which are with small sample sizes or insignificant research results, are unlikely to be published in academic journals (Viechtbauer, 2007). To evaluate publication bias within the current meta-analysis, we employed Egger’s regression test (Egger et al., 1997) and Begg’s funnel plots (Begg & Mazumdar, 1994).

### 2.6. Effect Size Computation and Analysis

Due to different studies measuring different indicators of well-being and using different scales, standardized mean difference was used to combine the statistics in this study to eliminate the influence of absolute values and unit differences in multiple studies. In addition, a 95% confidence interval (CI) is used to estimate that the sample statistic has a 95% probability of falling into the population parameter. Therefore, the standard mean difference and 95% CI were used in this meta-analysis. Cohen’s *d* was employed to calculate the effect size. In the *t*-test for the significance of the difference between the means of two independent groups, *d* represented the ratio of the difference between the means of the intervention group and the control group to the pooled standard deviation of both groups.

$$d=\frac{M_{i}-M_{c}}{S_{w}}$$

*d* represents the effect size, *M_i_* represents the mean of the intervention group, *M_c_* represents the mean of the control group, and *S_w_* refers to the pooled standard deviation within the intervention group and the control group, which is calculated using the following formula:

$$S_{w}=\sqrt{\frac{\left(n_{i}-1\right){\left(S_{i}\right)}^{2}+\left(n_{c}-1\right){\left(S_{c}\right)}^{2}}{n_{i}+n_{c}-2}}$$

According to Cohen (2013), an effect size 0.2 <= *d* < 0.5 is considered small, 0.5 <= *d* < 0.8 is considered medium, and *d* >= 0.8 is considered large. If SMD demonstrates a positive effect on well-being, the effect size is positive, and if it has a negative effect, the effect size is negative. To maintain consistency in the data, where positive values signify beneficial effects and negative values denote adverse effects, negative indicators were reversed in this study.

In this study, the statistical software Stata18 was employed for conducting the meta-analysis. To evaluate the variability in effect sizes across various studies, a heterogeneity test (Q test) was performed. If the effects are heterogeneous, a random-effects model should be used. Conversely, if the hypothesis of homogeneity is not rejected, a fixed-effects model should be used (Hedges & Vevea, 1998). Additionally, values of I^2^ are also a test for heterogeneity. When I^2^ = 0, it means no heterogeneity, and the larger the I^2^, the greater the heterogeneity. The degree of heterogeneity can be categorized as low, medium, and high, corresponding to I^2^ values of 25%, 50%, and 75% respectively (Higgins et al., 2003). Since heterogeneity was found among the included studies through the results, a random-effects model was considered the most suitable approach.

### 2.7. Moderation Analysis

Subgroup analysis and meta-regression analysis were conducted to determine whether the observed variances in effect sizes could be attributed to the presence of moderator variables. The subgroup analysis aimed to explore the potential moderating effects of these variables. To assess the existence of a moderating effect, the Q statistic and the I^2^ index of effect size were utilized as indicators. The Q statistical method, established by Hedges and Olkin (2014), was employed to evaluate the statistical significance of differences among the moderating variables. This method divides Q into two parts, Q_Within_ (Q_WIT_) and Q_Between_ (Q_BET_). While Q_WIT_ assesses homogeneity within the moderator variable, Q_BET_ evaluates homogeneity between the groups. Additionally, the meta-regression analysis was performed to investigate the moderating effects of age, gender, and detox duration.

## 3. Results

Finally, this meta-analysis included 20 studies with 56 effect sizes. Among them, 17 studies involved the positive indicators of well-being (k = 33), while 12 studies involved the negative indicators of well-being (k = 23). The meta-analysis comprised 10,106 participants, with sample sizes ranging from 38 to 888 participants. The mean age of the participants was 28.82 years. The number of independent samples reported per study varied from 1 to 6. Of the 20 studies, 13 were conducted in individualistic cultures, 5 in collectivistic cultures, and 1 in mixed culture countries. To explore the correlation between SMD and well-being, average effect sizes were calculated employing a random-effects model.

### 3.1. Publication Bias

**Figure 2:**
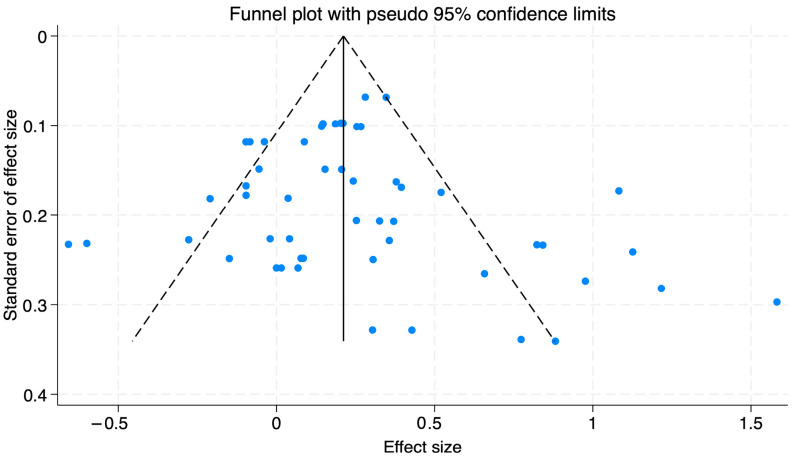
Funnel plot.

In this study, we employed two methods to identify this study’s publication bias. Initially, we utilized a funnel plot for all the studies to evaluate potential bias visually. The distribution of effect sizes from the 20 included studies displayed a symmetrical shape (see Figure 2), characteristic of nonbiased meta-analytic datasets. This symmetry indicated that the literature included in this study did not have publication bias.

Acknowledging the nature of funnel plot interpretation, Egger’s regression test was further conducted for more objective. The results are shown in Table 1. According to Egger’s test results, the intercept and *p*-values for overall well-being, positive indicators, and negative indicators are 0.726 (*p* > 0.05), 0.387 (*p* > 0.05), and 1.413 (*p* > 0.05), respectively. These findings further support the absence of publication bias, thereby reinforcing the validity of this meta-analysis.

### 3.2. Heterogeneity Test

A heterogeneity test was conducted following the calculation of effect sizes for each included study. The Q, I^2^, and τ^2^ statistics were computed to assess heterogeneity among the effect sizes, with the results detailed in Table 2. The findings showed that the Q value for overall well-being (Q = 179.32, *p* < 0.0001), positive indicators (Q = 103.40, *p* < 0.0001), and negative indicators (Q = 75.76, *p* < 0.0001) were statistically significant, suggesting heterogeneous effect sizes across the studies. The I^2^ statistic, which complements the Q statistic and allows for the determination of the degree of heterogeneity, was calculated as I^2^_overall_ = 69.3%, I^2^_positive_ = 69.1%, and I^2^_negative_ = 71.0%. According to Higgins et al. (2003), these values above 50% indicate a medium level of heterogeneity among the included studies. Therefore, the results of heterogeneity tests indicate that the random effects model employed in this study was correct and appropriate.

### 3.3. Overall Results

Utilizing a random effects model, this study investigated the overall effect of SMD on well-being. The analysis identified a total of 56 independent effect sizes of SMD on well-being, including 10,106 participants. Details regarding the common effect size, alongside its 95% confidence interval (lower and upper limits), as well as the z-values and *p*-values, are summarized in Table 3. The common effect size value for overall well-being was estimated at 0.233, with a 95% confidence interval ranging from 0.154 to 0.313. This effect size was found to be statistically significant (z (56) = 5.742, *p* < 0.0001), supporting H1. As seen in Table 3, we also found a very small mean positive effect of overall SMD on positive indicators of well-being (d = 0.231, z = 4.144, *p* < 0.0001, 95% CI[0.122, 0.340]), supporting H1a, and a very small mean positive effect on negative indicators of well-being (d = 0.239, z = 3.892, *p* < 0.0001, 95% CI[0.118, 0.359]), supporting H1b. These findings indicate that the correlation between SMD and well-being was positive and small. According to Ferguson’s (2009) recommendation, it was concluded that the relationship between SMD and well-being possesses a modest effect size.

**Figure 3:**
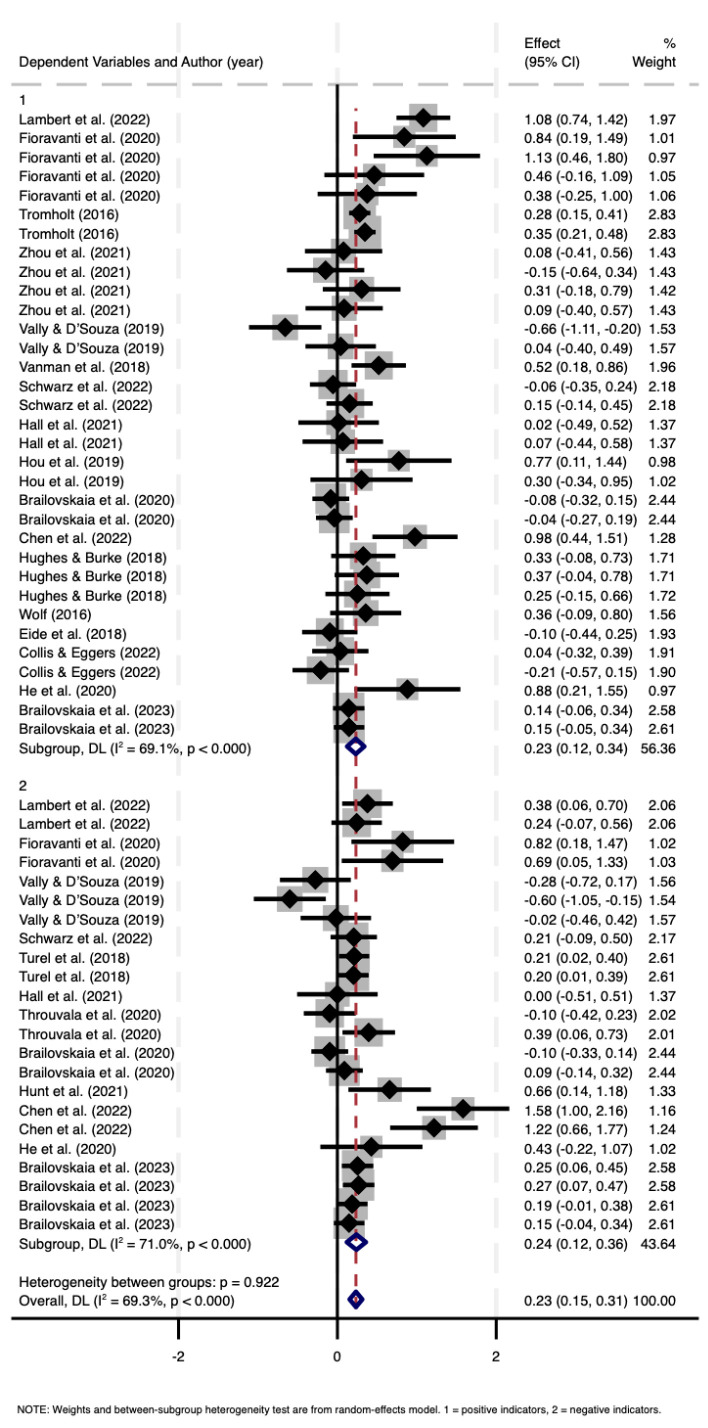
Forest plot according to random effects model (Eide et al., 2018; He et al., 2020; Lambert et al., 2022; Throuvala et al., 2020; Wolf, 2016).

Figure 3 presents a forest plot displaying the 56 independent effect sizes included in this meta-analysis, calculated as weighted averages within a 95% confidence interval. And, in the graph, the effect sizes from individual studies are represented by solid rhombus, while the horizontal lines extending from each solid rhombus denote the 95% confidence intervals associated with those effect sizes. The overall effect size of the study is represented by hollow rhombus. Red dashed line indicates the meta-analysis result. The intersection of this dashed line with the x-axis represents the common effect estimate. As seen in Figure 3, the calculated effect sizes for the relationship between SMD and overall well-being varied between −0.66 and 1.58. Furthermore, the effect sizes of positive indicators varied between −0.66 and 1.13, and the effect sizes of negative indicators varied between −0.60 and 1.58. The significance levels associated with these effects were confirmed, with the Q test indicating no significant difference between the mean correlations related to positive and negative well-being indicators (Q_BET_ = 0.01, *p* = 0.922). This suggests that SMD can increase the positive well-being indicators and reduce the negative emotions that affect well-being.

### 3.4. Moderator Analysis

The heterogeneity test was significant for overall well-being, positive indicators, and negative indicators, indicating potential moderators. To explore the sources of this heterogeneity, we conducted moderator analyses, including a subgroup analysis and meta-regression. The findings from these moderator analyses, which assess whether the effect size of the relationship between SMD and well-being varies according to factors such as age, gender, cultural background, detox types, and detox duration, are detailed in Table 4 and Table 5.

Age: The meta-regression results displayed in Table 5 indicate that age did not significantly impact the common effect size for overall well-being (B = 0.018, *p* = 0.122). Consequently, it can be concluded that participants’ age did not have a meaningful impact on the relationship between SMD and well-being. Accordingly, age was not identified as an important source of heterogeneity, leading to the conclusion that H2 was not supported. Moreover, the results further showed that age did not significantly affect the relationship between SMD and the positive indicators of well-being (B = 0.011, *p* = 0.538) and negative indicators (B = 0.027, *p* = 0.098).

Gender: In terms of gender, the meta-regression analysis revealed that the ratio of females within study samples did not significantly affect the overall effect size for well-being (B = 0.001, *p* = 0.998). Consequently, gender ratio was not identified as a notable source of heterogeneity in the studies examining this relationship, indicating that H3 was not supported. Furthermore, the analysis revealed that gender did not significantly affect the relationship between SMD and the positive indicators of well-being (B = 0.009, *p* = 0.978) or negative indicators (B = −0.011, *p* = 0.980).

Cultural Background: Upon examining the findings related to the moderator variable of cultural background, it was observed that the common effect size of overall well-being was 0.202, 95% CI [0.131, 0.272] for individualism; 0.287, 95% CI [−0.019, 0.594] for collectivism; and 0.710, 95% CI [0.447, 0.972] for mixed culture. Furthermore, a significant difference across these group effect sizes was detected (Q_BET_ = 13.55, *p* = 0.001). This result suggests that the effect size in mixed-culture countries is the highest, indicating that H4 was not supported.

Additionally, the mean effect of SMD on the positive indicators of well-being was significantly different (Q_BET_ = 6.69, *p* = 0.035), with effect sizes of 0.217, 95% CI [0.096, 0.337] for individualism; 0.233, 95% CI [−0.078, 0.544] for collectivism; and 0.687, 95% CI [0.350, 1.025] for mixed culture. Similarly, the relationship between SMD and negative indicators of well-being exhibited significant differences across the three subgroups (Q_BET_ = 6.18, *p* = 0.046), with effect sizes of 0.186, 95% CI [0.114, 0.257] for individualism; 0.372, 95% CI [−0.301, 1.046] for collectivism; and 0.757, 95% CI [0.303, 1.212] for mixed culture.

Detox Types: Upon analyzing the findings related to the moderator variable of detox types, it was found that there were no significant differences in the effect sizes for overall well-being among the groups categorized by full abstinence (0.223, 95% CI [0.120, 0.326]), limited use (0.121, 95% CI [0.019, 0.223]) and the therapy-based intervention (0.472, 95% CI [0.156, 0.788]) groups. The different types of detox did not significantly influence the correlation between SMD and well-being (Q_BET_ = 5.19, *p* = 0.074). Thus, detox type was not identified as a notable source of heterogeneity, indicating that H5 was not supported.

Regarding the relationship between SMD and the positive indicators of well-being, no significant differences were observed across the three intervention type groups (Q_BET_ = 2.63, *p* = 0.268). The effect sizes were 0.266, 95% CI [0.107, 0.424] for full abstinence; 0.110, 95% CI [−0.040, 0.259] for limited use; and 0.314, 95% CI [0.018, 0.610] for therapy-based intervention. Similarly, for the negative indicators of well-being, the analysis revealed no significant differences in the common effect among the three intervention groups (Q_BET_ = 2.70, *p* = 0.259). The effect sizes were 0.180, 95% CI [0.053, 0.307] for full abstinence; 0.142, 95% CI [−0.004, 0.289] for limited use; and 0.740, 95% CI [0.040, 1.439] for therapy-based intervention.

Intervention Duration: The findings from the meta-regression analysis assessing the moderator variable of detox duration revealed that this factor did not exert a significant influence on the overall effect size for well-being (B = 0.001, *p* = 0.785). Consequently, it can be concluded that the duration of the intervention did not influence the correlation between SMD and well-being. Thus, detox duration was an insignificant source of heterogeneity, indicating that H6 was not supported.

Furthermore, the analysis demonstrated that the moderation coefficient for intervention duration did not show statistical significance concerning the relationship between SMD and the positive indicators of well-being (B = −0.005, *p* = 0.302). In contrast, intervention duration did appear to moderate the relationship with the negative indicators of well-being (B = 0.032, *p* = 0.001), suggesting that as the proportion of female participants increased, a stronger effect on negative indicators was observed.

In conclusion, according to Table 4 and Table 5, age, gender, detox types, and detox duration did not significantly influence the heterogeneity of the effect size of overall well-being. Cultural background emerged as the primary source of heterogeneity. In terms of the positive indicators of well-being, cultural background was also the main source of heterogeneity. Further, for the negative indicators of well-being, cultural background and detox duration were the main sources of heterogeneity.

## 4. Discussion

With social media increasingly embedded in people’s lives and having a significant impact on their well-being, it makes sense to clarify the relationship between social media and well-being. Although previous meta-analyses have investigated the relationship and reported that there was no effect or only a small negative effect of social media on well-being (Huang, 2017; Wong et al., 2024), these studies mainly focus on the impact of social media usage behaviors. However, with the growing negative impact of social media, it is important to investigate the impact of social media discontinuous usage behaviors on mental health and well-being. This study aimed to statistically integrate the effect sizes from research examining the relationship between SMD and well-being through a meta-analysis. To this end, the results of 20 articles meeting the inclusion criteria were synthesized. The findings demonstrated that SMD affected well-being, influencing both positive and negative indicators. Additionally, these small correlations depend on cultural background and detox duration.

### 4.1. Main Effect

Using a random effects model, we estimated the common effect sizes for overall well-being, the positive indicators of well-being, and the negative indicators of well-being, yielding values of 0.233, 0.231, and 0.239, respectively. These results indicate that the impact of SMD on well-being was small but positive, increasing positive feelings and reducing negative feelings to improve overall well-being. These feelings are consistent with those of most studies (Brailovskaia et al., 2020; Chen et al., 2022; Hou et al., 2019; Hunt et al., 2021; Turel et al., 2018). Our results support some arguments based on the presumption of balance, suggesting that SMD is a response to the experience of temporal overload and invasion, and serves as a strategy to improve health and mindful presence (Syvertsen & Enli, 2020). The results showed that SMD can increase the positive indicators of well-being. SMD allows users to pay more attention to other activities, which can improve their well-being. Allcott et al. (2020) pointed out that deactivating social media not only reduced online engagement but also encouraged increased offline activities. For example, they can watch television and spend time with family and friends. SMD allows people to essentially spend more time interacting with members inside or outside the family (Coyne & Woodruff, 2023). Moreover, physical activities, like jogging, rose following SMD intervention, leading to a healthier lifestyle, greater life satisfaction, and reduced depression (Brailovskaia et al., 2020). Additionally, our results also showed that SMD can reduce the negative indicators of well-being, which are identical to the results of another meta-analysis (Ramadhan et al., 2024). Ramadhan et al. (2024) suggested that SMD can mitigate the factors that contribute to depressive symptoms in the digital realm, including exposure to negative social comparisons, experiences of cyberbullying, and information overload. Furthermore, social media addiction is recognized as a non-negligible contributor to negative emotions. SMD can effectively mitigate social media addiction through cognitive consequences and supporting techniques, which have the potential to improve mental health (Hou et al., 2019). Additionally, SMD can reduce perceived stress, particularly in excessive users (Turel et al., 2018).

However, this study is inconsistent with the results of Vally and D’Souza (2019) and Allcott et al. (2020), who held the opinion that SMD had a negative effect on well-being. Social media platforms, such as Facebook, serve as sources of entertainment, venues for organizing charitable or activist initiatives, and essential lifelines for individuals who may feel socially isolated, thereby enhancing their quality of life (Allcott et al., 2020). Thus, SMD may cause individuals to derive less pleasure from it and experience more negative emotions. These adverse effects are likely influenced by the specific functions for which social media is utilized (Vally & D’Souza, 2019). Given the fundamental role of social media in facilitating social connections (Harari & Gosling, 2016), SMD may be perceived as distancing oneself from a vital source of interaction.

### 4.2. Moderator Analysis

Another finding of this meta-analysis is the observed heterogeneity in effect sizes among the included studies. To investigate the sources of this heterogeneity, we performed moderator analyses on various potential moderating variables, including age, gender, cultural background, detox types, and duration, to explore the source of this heterogeneity. The findings indicated that only cultural background accounted for significant variability in the correlation between SMD and overall well-being. Additionally, while detox duration did not influence the relationship between SMD and overall well-being (including both positive and negative indicators), it did moderate the relationship with negative indicators specifically. It was determined that the other potential moderators examined did not serve as significant sources of variability in differentiating the effect of SMD on well-being.

#### 4.2.1. Moderating Effect of Age and Gender

Age did not serve as a moderating factor in the relationship between SMD and well-being. A possible explanation for this is the limited age range represented in the included studies, which may have restricted our ability to analyze the varying effects of SMD across different age demographics. Most of the included studies focused on college students, with relatively little research addressing adolescents or older adults. Of the 20 studies, 12 involved college students. Additionally, this finding indicates that although different age groups utilize social media for distinct purposes, the psychological benefits of SMD remain largely consistent across various ages. Research indicates that teenagers tend to prioritize making new connections and engage in more frequent and rapid posting, whereas older adults are inclined to create longer posts and focus on family-related discussions (Chang et al., 2015; Pfeil et al., 2009). Consequently, despite the generational variations in social media usage patterns, the psychological health benefits of engaging in detox practices are relevant and beneficial for individuals across all age demographics.

Similarly, gender did not moderate the relationship between SMD and well-being. This result might be attributed to the increasing ubiquity of social media as a global communication platform, equally accessible and influential for both genders (Krasnova et al., 2017). Consequently, the universality of social media for both genders may reduce the differences in the effect of SMD on them. However, this result contrasts with the findings of Fioravanti et al. (2020), who argued that women experienced greater levels of life satisfaction and positive affect following social media abstinence, with no such effects observed among men. This discrepancy can be explained by differences in appearance comparison between females and males. Women may be more influenced by the portrayal of physical appearance on social media, resulting in a stronger emotional response post-detox. Furthermore, the study by Fioravanti et al. (2020) focused more on well-being related to physical appearance comparison, whereas our meta-analysis evaluated the impact of SMD on well-being from a broader perspective. This comprehensive viewpoint may account for the observed universality of gender effects in our findings, as it encompassed a wider range of well-being indicators rather than just those tied to body image or physical appearance.

#### 4.2.2. Moderating Effect of Culture Background

Our analysis revealed that cultural background moderates the relationship between SMD and overall well-being, encompassing both its positive and negative dimensions. Unexpectedly, the effect of SMD on well-being was most pronounced in the samples from mixed cultural contexts, which was not consistent with our hypothesis. In these mixed cultural environments, SMD was associated with greater improvements in both positive and negative emotions. A possible reason might be that people in mixed-cultural countries have complex cultural orientations that combine individualism and collectivism. SMD can relieve them of group stress without causing too much anxiety. Furthermore, compared with individualist cultures, the mean effect is stronger in the samples from collectivist cultures. Previous research has demonstrated that in collectivist countries, interdependent well-being is often viewed through a lens of interpersonal relationships, emphasizing interdependence (Hitokoto & Uchida, 2015; Lu & Gilmour, 2006). Individuals in these cultures may face heightened stress from social media due to norms of reciprocity and the demands of maintaining extensive social networks (Jiang, 2022). The SMD could relieve them of this stress, thereby improving their well-being. Consequently, the influence of SMD on well-being is stronger in collectivist countries compared to individualist countries.

#### 4.2.3. Moderating Effect of Detox Types and Duration

Detox types did not moderate the relationship between SMD and well-being. A possible explanation for this is that all types of SMD interventions could affect well-being. Most studies included in this meta-analysis focused on the full abstinence or limited use approaches (Hunt et al., 2021; Schwarz et al., 2022; Turel et al., 2018), which have demonstrated a positive influence on well-being. Moreover, there are other therapy methods. Grayscaling has been identified as effective in reducing screen time and social media use, thereby enhancing well-being (Dekker & Baumgartner, 2023). This method can increase users’ perceived control over their phone use and mitigate feelings of overuse, online vigilance, and related stress. Similarly, Stair Transformational Systemic Therapy (STST) has proven effective in addressing the problem of social media use, violence, and loneliness while boosting social support and improving depressive (Chen et al., 2022). Therefore, whether through full abstinence, limited use, or therapy-based interventions, all the detox approaches positively impact users’ well-being with minimal differences in effectiveness. However, this result contrasts with the findings of Plackett et al. (2023), who conducted a systematic review that indicated therapy-based interventions were more effective than the other two types. Their conclusions were drawn from the proportion of the literature reporting the significance of effects associated with various SMD types, revealing that 85% of the studies focused on therapy-based interventions demonstrated significant positive outcomes, compared to 25% for full abstinence and 20% for limited use interventions. While their research methodology is compelling from a probabilistic standpoint, the potential for bias remains a concern. In contrast, our study employs a meta-analytic approach, a robust statistical technique that synthesizes findings from multiple social science studies (Glass, 1976). This method enables a more objective analysis of the effects of SMD on well-being, as well as the moderating influences of different variables. It is important to note that varying analytical methods can yield inconsistent results. Furthermore, our findings can be further contextualized by examining the role of psychological adaptation. Different detox strategies may drive individuals to adopt diverse coping mechanisms in response to the negative emotions often associated with social media use. Whether through full abstinence, limited use, or therapy-based interventions, individuals can mitigate these adverse effects, thereby enhancing their overall well-being. Research by Reinecke et al. (2022) highlights the crucial role of emotional regulation in the link between social media use and subjective well-being. This underscores the notion that the SMD process may foster a more conscious reflection on social media habits, ultimately leading to improvements in mental health and overall well-being.

Detox duration did not moderate the effect of SMD on the overall level of well-being and its positive indicators. While less time spent on smartphones and social media could lead to increased well-being, full abstinence from social media is not necessarily required (Brailovskaia et al., 2023). Even a short-term SMD can liberate individuals from the adverse effects of social media, providing them with psychological relief and recovery. Further, the psychological effects of SMD may also lie in the enhancement of individuals’ self-awareness. The improvement in self-regulation is related to individuals’ psychological adaptability and coping strategies rather than the duration of the detox itself. Research indicates that increased self-awareness can help individuals effectively identify and regulate their emotions, thereby improving mental health (Wagani & Gaur, 2024). However, we found that intervention duration moderated the relationship between SMD and the negative indicators of well-being. The results showed that the duration of the intervention is positively correlated with the effectiveness of SMD in enhancing the outcomes related to negative affects. The principle of intervention on addictive behavior may explain this result. Following an initial phase characterized by heightened cravings, withdrawal, and relapse, symptoms of addiction typically diminish (Wetterling et al., 1996). At the beginning of SMD, users might experience negative emotions due to cravings, but as the intervention time grows, the negative emotions are alleviated. Therefore, the longer the intervention period, the greater the impact of SMD on negative indicators of well-being.

### 4.3. Theoretical and Practical Implications

This study offers several significant theoretical implications. First, by employing a meta-analysis approach, we synthesized evidence from various randomized controlled trials on the effect of SMD on well-being. Our analysis elucidates how SMD contributes to different kinds of indicators of well-being, ultimately impacting overall well-being. The application of meta-analysis effectively addresses the statistical limitations often associated with small-sample empirical studies. Consequently, we traced and summarized the relationship between SMD and well-being from a macro and comprehensive perspective. Additionally, we expanded the application of UGT and analyzed the impact of “no use” behavior on different indicators of gratifications. Moreover, this study synthesizes findings from randomized controlled trials, which can address causality in the relationship between SMD and well-being. Temporality and causality can be explored by longitudinal and experimental methods (Hartanto et al., 2021). Therefore, unlike the prior literature, which relied heavily on correlational studies and faced challenges such as reverse causation, this study allows for stronger inferences regarding causality and the directionality of the relationship between SMD and well-being. Furthermore, this meta-analysis identifies various moderators that influence the relationships between SMD and well-being. Our results suggest that culture background and intervention duration contribute to the heterogeneity observed. Nonetheless, the specific mechanisms underlying these moderators warrant further exploration in future empirical research.

Social media has become deeply integrated into various aspects of daily life, resulting in an increasing number of individuals experiencing fatigue due to overload and psychosocial stress. Consequently, understanding the effect of SMD on well-being holds significant practical implications for users, social media platforms, and governments. For users, although the effect is small, in a certain size of the population, this small increase may have a cumulative effect. For example, a weekly SMD may have a positive impact on an individual’s overall well-being over a long period of time. In addition, small effects may be amplified in people who are more stressed or show signs of addiction to social media use, resulting in significant improvements in their psychological state. For social media platforms, it is recommended to establish a social media overuse monitoring function. When users spend too much time on social media, the platform can alert users and suggest they carry out a short SMD. For the government, public health is always a priority. Even if the effect is small, SMDs that can be universally advocated and promoted may have a cumulative positive effect on the mental health of the entire community. Governments can introduce SMD policies or calls to promote public health.

### 4.4. Limitations and Implications for Future Research

Our meta-analysis not only examined the intricate relationship between SMD and well-being but also incorporated both positive and negative well-being indicators. Additionally, we highlighted the moderating role of cultural background in this correlation. Although our study offered a quantitative synthesis of the connection between SMD and well-being, it is significant to note that the results are constrained by the scope of the existing research in this domain. Our analysis revealed notable gaps in the current literature, calling the need for further investigation along several promising avenues.

The current meta-analysis is primarily constrained to quantitative research investigating the relationship between SMD and well-being. To gain a deeper understanding of individuals’ perceptions regarding SMD, it is necessary initially to conduct qualitative research. Moreover, the studies included in this meta-analysis predominantly focused on young individuals and college students, resulting in findings that lack broader representativeness. Future research should explore similar or comparative studies across diverse age groups, including adolescents and older adults. In the current study, except for cultural background and intervention duration, the other variables did not demonstrate statistical significance in accounting for the heterogeneity. Consequently, future meta-analyses examining the correlation between SMD and well-being should consider a wider range of moderating variables. Further, the incorporation of new studies into the literature will enhance the potential for subsequent meta-analyses on this topic.

## 5. Conclusions

The empirical studies surrounding the correlation between SMD and well-being are notably inconsistent. In our study, we conducted a meta-analysis to statistically assess this correlation and to identify any potential moderators that may influence it. Overall, the effect size of this relationship is small and varies based on cultural background and the duration of the intervention. Specifically, the association between SMD and well-being tends to be more favorable in mixed cultural contexts. Furthermore, longer intervention periods are associated with greater improvements in negative indicators of well-being. Consequently, we suggest that users can effectively engage in SMD to mitigate the adverse effects associated with social media usage.

## Figures and Tables

**Table 1:** Results of Egger’s Regression Test.

Dependent Variable	k	Egger’s Intercept	SE	95% CI		*p*
				LL	UL	
Overall well-being	56	0.726	0.543	−0.362	1.813	0.187
Positive indicators	33	0.387	0.670	−0.981	1.754	0.568
Negative indicators	23	1.413	0.958	−0.580	3.405	0.155

Note: k = number of studies; SE = Standard Error; CI = confidence interval; LL = lower limit; UL = upper limit.

**Table 2:** Results of heterogeneity test.

Dependent Variable	Q	df	*p*	I^2^	τ^2^
Overall well-being	179.32	55	0.000	69.3	0.054
Positive indicators	103.40	32	0.000	69.1	0.059
Negative indicators	75.76	22	0.000	71.0	0.053

Note: Q = Cochran’s measure of homogeneity; I^2^ = proportion of total variance in population effect size; τ^2^ = tau-square.

**Table 3:** Effect size according to random effects model.

Dependent Variable	k	N	ES	z	*p*	95% CI	
						LL	UL
Overall well-being	56	10,106	0.233	5.742	0.000	0.154	0.313
Positive Indicators	33	5428	0.231	4.144	0.000	0.122	0.340
Negative Indicators	23	4678	0.239	3.892	0.000	0.118	0.359

Note: k = number of studies; N = total number of participants for all the studies combined; ES = effect size; z = z-value; CI = confidence interval; LL = lower limit; UL = upper limit.

**Table 4:** Results of moderator analysis (subgroup analysis).

Dependent Variable	Moderator Variable	Category	k	ES	95% CI		Q_b_	df	*p*
					Lower	Upper			
Overall well-being	Culture Background	Individualism	32	0.202	0.131	0.272	13.55	2	0.001
		Collectivism	16	0.287	−0.019	0.594			
		Mixed Culture	6	0.710	0.447	0.972			
	Intervention Type	Full Abstinence	30	0.223	0.120	0.326	5.19	2	0.074
		Limited Use	15	0.121	0.019	0.223			
		Therapy-based	11	0.472	0.156	0.788			
Positive Indicators	Culture Background	Individualism	17	0.217	0.096	0.337	6.69	2	0.035
		Collectivism	10	0.233	−0.078	0.544			
		Mixed Culture	4	0.687	0.350	1.025			
	Intervention Type	Full Abstinence	17	0.266	0.107	0.424	2.63	2	0.268
		Limited Use	9	0.110	−0.040	0.259			
		Therapy-based	7	0.314	0.018	0.610			
Negative Indicators	Culture Background	Individualism	15	0.186	0.114	0.257	6.18	2	0.046
		Collectivism	6	0.372	−0.301	1.046			
		Mixed Culture	2	0.757	0.303	1.212			
	Intervention Type	Full Abstinence	13	0.180	0.053	0.307	2.70	2	0.259
		Limited Use	6	0.142	−0.004	0.289			
		Therapy-based	4	0.740	0.040	1.439			

Note: k = number of studies; ES = effect size; CI = confidence interval.

**Table 5:** Results of moderator analysis (meta-regression).

Dependent Variable	Moderator Variable	k	B	SE	Z	*p*	95% CI	
							LL	UL
Overall well-being	Age	49	0.018	0.012	1.55	0.122	−0.005	0.042
	Gender	56	0.001	0.259	0.00	0.998	−0.507	0.508
	Intervention Duration	56	0.001	0.004	0.27	0.785	−0.007	0.009
Positive Indicators	Age	29	0.011	0.018	0.62	0.538	−0.024	0.045
	Gender	33	0.009	0.333	0.03	0.978	−0.643	0.661
	Intervention Duration	33	−0.005	0.004	−1.03	0.302	−0.013	0.004
Negative Indicators	Age	20	0.027	0.017	1.65	0.098	−0.005	0.060
	Gender	23	−0.011	0.441	−0.02	0.980	−0.875	0.853
	Intervention Duration	23	0.032	0.009	3.43	0.001	0.014	0.051

Note: k = number of studies; B = regression included; SE = Standard Error; Z = Z-value; CI = confidence interval; LL = lower limit; UL = upper limit.

## Data Availability

The data that support the findings of this study are available upon request from the corresponding author.

## Supplementary Materials

The following supporting information can be downloaded at https://www.mdpi.com/article/10.3390/bs15030290/s1, Table S1: References of included studies; Table S2: Characteristics of included studies.

## Abbreviations

The following abbreviations are used in this manuscript:

SMD	social media detox
RCT	randomized controlled trial
UGT	Uses and Gratifications Theory
