# Supplementary material for: Am I Happier Without You? Social Media Detox and Well-Being: A Meta-Analysis of Randomized Controlled Trials
Source: Behav Sci (Basel). 2025 Mar 1;15(3):290. doi: 10.3390/bs15030290 (PMC11939267; doi:10.3390/bs15030290)
Supplement: Supplementary file 1 [file behavsci-15-00290-s001.zip › behavsci-3443976_Supplementary T1 References.pdf]

References of Included Studies

Brailovskaia, J., Delveaux, J., John, J., Wicker, V., Noveski, A., Kim, S., Schillack, H., & Margraf, J. (2023). Finding the "sweet spot" of smartphone use: Reduction or abstinence to increase well-being and healthy lifestyle?! An experimental intervention study. *Journal of Experimental Psychology: Applied*, *29*(1), 149. https://doi.org/10.1037/xap0000430

Brailovskaia, J., Ströse, F., Schillack, H., & Margraf, J. (2020). Less Facebook use—More well-being and a healthier lifestyle? An experimental intervention study. *Computers in Human Behavior*, *108*, 106332. https://doi.org/10.1016/j.chb.2020.106332

Chen, Y., Liu, X., Chiu, D. T., Li, Y., Mi, B., Zhang, Y., Ma, L., & Yan, H. (2022). Problematic Social Media Use and Depressive Outcomes among College Students in China: Observational and Experimental Findings. *International Journal of Environmental Research and Public Health*, *19*(9), 4937. https://doi.org/10.3390/ijerph19094937

Collis, A., & Eggers, F. (2022). Effects of restricting social media usage on wellbeing and performance: A randomized control trial among students. *PLoS ONE*, *17*(8), e0272416. https://doi.org/10.1371/journal.pone.0272416

Eide, T. A., Aarestad, S. H., Andreassen, C. S., Bilder, R. M., & Pallesen, S. (2018). Smartphone restriction and its effect on subjective withdrawal related scores. *Frontiers in Psychology*, *9*, 1444. https://doi.org/10.3389/fpsyg.2018.01444

Fioravanti, G., Prostamo, A., & Casale, S. (2020). Taking a Short Break from Instagram: The Effects on Subjective Well-Being. *Cyberpsychology, Behavior, and Social Networking*, *23*(2), 107–112. https://doi.org/10.1089/cyber.2019.0400

Hall, J. A., Xing, C., Ross, E. M., & Johnson, R. M. (2021). Experimentally manipulating social media abstinence: Results of a four-week diary study. *Media Psychology*, *24*(2), 259–275. https://doi.org/10.1080/15213269.2019.1688171

He, J.-w., Tu, Z.-h., Xiao, L., Su, T., & Tang, Y.-x. (2020). Effect of restricting bedtime mobile phone use on sleep, arousal, mood, and working memory: A randomized

pilot trial. *PLoS ONE*, *15*(2), e0228756. https://doi.org/10.1371/journal.pone.0228756

Hou, Y., Xiong, D., Jiang, T., Song, L., & Wang, Q. (2019). Social media addiction: Its impact, mediation, and intervention. *Cyberpsychology: Journal of Psychosocial Research on Cyberspace*, *13*(1), 4. https://doi.org/10.5817/CP2019-1-4

Hughes, N., & Burke, J. (2018). Sleeping with the frenemy: How restricting 'bedroom use'of smartphones impacts happiness and wellbeing. *Computers in Human Behavior*, *85*, 236–244. https://doi.org/10.1016/j.chb.2018.03.047

Hunt, M., All, K., Burns, B., & Li, K. (2021). Too Much of a Good Thing: Who We Follow, What We Do, And How Much Time We Spend on Social Media Affects Well-Being. *Journal of Social and Clinical Psychology*, *40*(1), 46–68. https://doi.org/10.1521/jscp.2021.40.1.46

Lambert, J., Barnstable, G., Minter, E., Cooper, J., & McEwan, D. (2022). Taking a one-week break from social media improves well-being, depression, and anxiety: A randomized controlled trial. *Cyberpsychology, Behavior, and Social Networking*, *25*(5), 287–293. https://doi.org/https://doi.org/10.1089/cyber.2021.0324

Schwarz, D., Steinau, K., Kraus, L., & In-Albon, T. (2022). The Effect of a 1-Week Abstinence From Instagram on Mental Health in Youth and Young Adults. *Kindheit und Entwicklung*, *31*(4), 200–210. https://doi.org/10.1026/0942-5403/a000392.

Throuvala, M. A., Griffiths, M. D., Rennoldson, M., & Kuss, D. J. (2020). Mind over matter: Testing the efficacy of an online randomized controlled trial to reduce distraction from smartphone use. *International Journal of Environmental Research and Public Health*, *17*(13), 4842. https://doi.org/10.3390/ijerph17134842

Tromholt, M. (2016). The Facebook experiment: Quitting Facebook leads to higher levels of well-being. *Cyberpsychology, Behavior, and Social Networking*, *19*(11), 661–666. https://doi.org/10.1089/cyber.2016.0259

Turel, O., Cavagnaro, D. R., & Meshi, D. (2018). Short abstinence from online social networking sites reduces perceived stress, especially in excessive users.

Psychiatry Research, *270*, 947–953. https://doi.org/10.1016/j.psychres.2018.11.017

Vally, Z., & D’Souza, C. G. (2019). Abstinence from social media use, subjective well-being, stress, and loneliness. *Perspectives in Psychiatric Care*, *55*(4), 752–759. https://doi.org/10.1111/ppc.12431

Vanman, E. J., Baker, R., & Tobin, S. J. (2018). The burden of online friends: The effects of giving up Facebook on stress and well-being. *The Journal of Social Psychology*, *158*(4), 496–508. https://doi.org/10.1080/00224545.2018.1453467

Wolf, A. (2016). Facebook and mental wellbeing: A crossover randomised controlled study. *F1000Research*, *5*, 1311. https://doi.org/10.12688/f1000research.8835.1

Zhou, X., Rau, P. P., Yang, C. L., & Zhou, X. (2021). Cognitive Behavioral Therapy-Based Short-Term Abstinence Intervention for Problematic Social Media Use: Improved Well-Being and Underlying Mechanisms. *Psychiatric Quarterly*, *92*(2), 761–779. https://doi.org/10.1007/s11126-020-09852-0
