# Supplementary material for: Am I Happier Without You? Social Media Detox and Well-Being: A Meta-Analysis of Randomized Controlled Trials
Source: Behav Sci (Basel). 2025 Mar 1;15(3):290. doi: 10.3390/bs15030290 (PMC11939267; doi:10.3390/bs15030290)
Supplement: Supplementary file 1 [file behavsci-15-00290-s001.zip › behavsci-3443976_Supplementary T2 Characteristics of Included Studies.pdf]

Characteristics of Included Studies

Author	Year	Country	Number	Mean age	Proportion of female	Detox types	Detox duration	Indicators of SWB	N_I	$Mean_I$	SD_I	N_c	$Mean_c$	SD_c
Jeffrey Lambert	2022	US	154	29.6	62.00%	Full abstinence	1 week	Well-being	81	9.93	7.88	73	1.13	8.40
Jeffrey Lambert	2022	US	154	29.6	62.00%	Full abstinence	1 week	Depression	81	2.62	4.40	73	0.89	4.75
Jeffrey Lambert	2022	US	154	29.6	62.00%	Full abstinence	1 week	Anxiety	81	2.07	4.19	73	0.98	4.80
Giulia Fioravanti	2019	Italy	40	25.05	100.00%	Full abstinence	1 week	Life satisfaction	20	2.55	5.34	20	-2.85	7.33
Giulia Fioravanti	2019	Italy	40	25.05	100.00%	Full abstinence	1 week	Positive affect	20	4.60	7.30	20	-3.10	6.34
Giulia Fioravanti	2019	Italy	40	25.05	100.00%	Full abstinence	1 week	Negative affect	20	6.90	8.17	20	0.75	6.70
Giulia Fioravanti	2019	Italy	40	25.05	0.00%	Full abstinence	1 week	Life satisfaction	20	1.15	5.85	20	-1.30	4.64
Giulia Fioravanti	2019	Italy	40	25.05	0.00%	Full abstinence	1 week	Positive affect	20	1.15	6.22	20	-1.45	7.55
Giulia Fioravanti	2019	Italy	40	25.05	0.00%	Full abstinence	1 week	Negative affect	20	6.40	9.14	20	1.05	5.97
Morten Tromholt	2016	Denmark	888	34	86.00%	Full abstinence	1 week	Life satisfaction	516	8.11	1.23	372	7.74	1.43
Morten Tromholt	2016	Denmark	888	34	86.00%	Full abstinence	1 week	Emotions	516	36.21	6.09	372	33.99	6.81

Xingchen Zhou	2020	China	65	28.8	60.00%	Therapy-based interventions	1 week	Life satisfaction	33	0.40	1.06	32	0.32	1.00
Xingchen Zhou	2020	China	65	28.8	60.00%	Therapy-based interventions	1 week	Work satisfaction	33	-0.04	0.48	32	0.03	0.46
Xingchen Zhou	2020	China	65	28.8	60.00%	Therapy-based interventions	2 weeks	Life satisfaction	33	0.74	1.09	32	0.41	1.07
Xingchen Zhou	2020	China	65	28.8	60.00%	Therapy-based interventions	2 weeks	Work satisfaction	33	0.03	0.47	32	-0.01	0.47
Zahir Vally	2019	UAE	78	22.13	52.60%	Full abstinence	1 week	Subjective well-being	39	4.37	1.26	39	5.16	1.14
Zahir Vally	2019	UAE	78	22.13	52.60%	Full abstinence	1 week	Negative affect	39	-2.6	0.89	39	-2.36	0.84
Zahir Vally	2019	UAE	78	22.13	52.60%	Full abstinence	1 week	Positive affect	39	3.34	0.63	39	3.31	0.81
Zahir Vally	2019	UAE	78	22.13	52.60%	Full abstinence	1 week	Loneliness	39	-3.81	1.3	39	-3.08	1.13
Zahir Vally	2019	UAE	78	22.13	52.60%	Full abstinence	1 week	Perceived stress	39	-1.79	0.49	39	-1.78	0.51
Eric Vanman	2018	Australia	138	22.43	63.00%	Full abstinence	5 days	Subjective well-being	60	1.85	4.97	78	-0.93	5.61
Daniela Schwarz	2023	Germany	185	22.8	76.20%	Full abstinence	1 week	Depression	79	0.19	0.45	106	0.09	0.51

Daniela Schwarz	2023	Germany	185	22.8	76.20%	Full abstinence	1 week	Self-esteem	79	0.08	0.46	106	0.11	0.60
Daniela Schwarz	2023	Germany	185	22.8	76.20%	Full abstinence	1 week	General mental state	79	0.57	1.48	106	0.33	1.62
Ofir Turel	2018	US	555	24.01	42.88%	Full abstinence	1 week	Absolute stress	413	0.72	1.04	142	0.50	1.06
Ofir Turel	2018	US	555	24.01	42.88%	Full abstinence	1 week	Relative stress	413	0.14	0.21	142	0.09	0.33
Jeffrey A. Hall	2019	US	61	26.8	78.50%	Full abstinence	4 weeks	Loneliness	26	-2.57	1.24	35	-2.57	1.17
Jeffrey A. Hall	2019	US	61	26.8	78.50%	Full abstinence	4 weeks	Affective well-being	26	4.81	1.31	35	4.79	1.31
Jeffrey A. Hall	2019	US	61	26.8	78.50%	Full abstinence	4 weeks	Quality of day	26	4.87	1.32	35	4.78	1.32
Melina A. Throuvala	2020	UK	143	20.72	82.00%	Therapy-based interventions	10 days	Stress	72	0.34	4.68	71	0.84	5.69
Melina A. Throuvala	2020	UK	143	20.72	82.00%	Therapy-based interventions	10 days	Anxiety	72	1.18	5.35	71	-0.81	4.71
Yubo Hou	2019	China	38	19.71	50.00%	Therapy-based interventions	2 weeks	Mental health	21	2.47	4.20	17	-0.83	4.35
Yubo Hou	2019	China	38	19.71	50.00%	Therapy-based interventions	2 weeks	Self-esteem	21	2.00	3.45	17	0.94	3.54

Julia Brailovskaia	2020	Germany	286	25.39	77.64%	Limited use	1 week	Life satisfaction	140	0.05	5.94	146	0.53	5.53
Julia Brailovskaia	2020	Germany	286	25.39	77.64%	Limited use	1 week	Depressive symptoms	140	0.52	4.79	146	0.98	4.74
Julia Brailovskaia	2020	Germany	286	25.39	77.64%	Limited use	2 weeks	Life satisfaction	140	0.55	6.11	146	0.77	5.40
Julia Brailovskaia	2020	Germany	286	25.39	77.64%	Limited use	2 weeks	Depressive symptoms	140	0.77	4.23	146	0.39	4.40
Melissa G. Hunt	2021	US	88	-	73.00%	Limited use	3 weeks	Depression	30	5.00	4.69	30	-4.63	20.17
Yonghua Chen	2022	China	60	-	66.67%	Therapy-based interventions	1 month	Depression	30	13.00	9.36	30	-2.47	10.18
Yonghua Chen	2022	China	60	-	66.67%	Therapy-based interventions	1 month	Loneliness	30	5.06	3.53	30	0.63	3.75
Yonghua Chen	2022	China	60	-	66.67%	Therapy-based interventions	1 month	Perceived social support	30	10.87	10.93	30	-1.07	13.40
Nicola Hughes	2018	UK	95	-	67.00%	Limited use	1 week	Subjective happiness	49	1.30	4.07	46	0.00	3.90
Nicola Hughes	2018	UK	95	-	67.00%	Limited use	1 week	Life satisfaction	49	3.80	11.48	46	-0.50	11.73
Nicola Hughes	2018	UK	95	-	67.00%	Limited use	1 week	Positive emotions	49	1.70	8.61	46	0.00	3.80
Achim Wolf	2016	UK	78	21	66.67%	Full	2 weeks	Mental well-	40	46.00	5.92	38	43.70	6.96

						abstinence		being						
Tine A. Eide	2018	Norway	127	25	72.40%	Full abstinence	3 days	Positive affect	67	2.57	0.67	60	2.64	0.79
Avinash Collis	2022	Europe	122	22.1	51.00%	Limited use	2 months	Life satisfaction	60	0.10	5.67	62	-0.10	5.20
Avinash Collis	2022	Europe	122	22.1	51.00%	Limited use	2 months	Subjective well-being	60	-0.60	2.71	62	0.00	3.00
Jing-wen He	2020	China	38	21	31.58%	Limited use	1 week	Positive affect	19	5.00	6.79	19	-0.58	5.83
Jing-wen He	2020	China	38	21	31.58%	Limited use	1 week	Negative affect	19	3.42	5.33	19	0.84	6.65
Julia Brailovskaia	2023	Germany	393	26.36	76.48%	Full abstinence	1 week	Life satisfaction	200	0.59	5.45	193	-0.17	5.20
Julia Brailovskaia	2023	Germany	393	26.36	76.48%	Full abstinence	1 week	Depression	200	0.99	3.93	193	0.02	3.70
Julia Brailovskaia	2023	Germany	393	26.36	76.48%	Full abstinence	1 week	Anxiety	200	1.01	3.47	193	0.07	3.57
Julia Brailovskaia	2023	Germany	419	26.36	76.48%	Limited use	1 week	Life satisfaction	226	0.60	5.29	193	-0.17	5.20
Julia Brailovskaia	2023	Germany	419	26.36	76.48%	Limited use	1 week	Depression	226	0.72	3.80	193	0.02	3.70
Julia Brailovskaia	2023	Germany	419	26.36	76.48%	Limited use	1 week	Anxiety	226	0.59	3.49	193	0.07	3.57
